# An atypical presentation of pyoderma gangrenosum with systemic features and pulmonary involvement in a toddler

**DOI:** 10.1186/1546-0096-9-S1-P35

**Published:** 2011-09-14

**Authors:** Despoina Maritsi, Christina Neila, Millie Konstantinidou, Elizabeth Dimitriou

**Affiliations:** 1 Department of General Pediatrics, Iaso Paediatric Hospital, Athens, Greece; 2 Department of Orthopedics, Iaso Paediatric Hospital, Athens, Greece

## Aim

To describe the first case of pyoderma gangrenosum (PG) with pulmonary involvement in the paediatric population.

## Methods

A 15 month old female presented with a red hot and painful indurated lesion on the left anterior tibial surface. She was treated intravenous antibiotics. The lesion erupted, creating a wide ulcer with violaceous borders. Pyrexia and malaise persisted, while inflammatory markers continued to rise besides antibiotic treatment. The lesion didn’t improve with repetitive debridement. Blood cultures, skin cultures including extended cultures for mycobacteria and fungi were negative. Her clinical condition deteriorated; she had weight loss, anemia, fever with rigors and mild tachypnoea. A chest x-ray showed diffuse interstitial involvement; HRCT showed cavitating lesions in upper and lower lobes bilaterally. Differential diagnosis included primary immunodeficiencies (including CGD), systemic vasculitides or an underlying hematological malignancy. Her humoral and cellular immune response and NBT were normal; p-ANCA and c-ANCA were negative, ACE was normal, repetitive fecal occult blood tests were negative; urinalysis was clear, BMA showed reactive changes in keeping with an inflammatory process. Skin biopsy showed findings consistent with pyoderma gangrenosum; there was no evidence of vasculitis or granulomas. She received high dose pulsed methylprednisolone followed by oral corticosteroids and six doses of IVIG at monthly intervals followed by azathioprine. She remains clinically well and her inflammatory markers have subsided.

## Discussion

Pyoderma gangrenosum is a rare ulcerative condition of unknown cause. The diagnosis is made by excluding other causes of similar lesions, including infection, malignancy, vasculitis, diabetes, and trauma. A systemic illness is identified in 50% of cases. Children may be affected, accounting for 3-4% of cases. Although highly infrequent in the absence of an underlying illness, extra-cutaneous involvement may include pulmonary, gastrointestinal and ocular findings. This case illustrates the first report of PG with systemic involvement in a toddler. Even if no other relevant symptom was present in our patient, PAPA syndrome needs to be considered in all cases of PG following exclusion of commoner diseases.

